# Elevated neutrophil-to-lymphocyte ratio and monocyte-to-lymphocyte ratio and decreased platelet-to-lymphocyte ratio are associated with poor prognosis in multiple myeloma

**DOI:** 10.18632/oncotarget.13320

**Published:** 2016-11-12

**Authors:** Lihui Shi, Xiaoqi Qin, Huijun Wang, Yonghui Xia, Yuanyuan Li, Xuejing Chen, Lei Shang, Yu-Tzu Tai, Xiaoyan Feng, Prakrati Acharya, Chirag Acharya, Yan Xu, Shuhui Deng, Mu Hao, Dehui Zou, Yaozhong Zhao, Kun Ru, Lugui Qiu, Gang An

**Affiliations:** ^1^ State Key Laboratory of Experimental Hematology, Institute of Hematology and Blood Diseases Hospital, Chinese Academy of Medical Science and Peking Union Medical College, Tianjin, China; ^2^ Department of Hematology, The Second Hospital of Shanxi Medical University, Taiyuan, China; ^3^ LeBow Institute for Myeloma Therapeutics and Jerome Lipper Center for Multiple Myeloma Center, Dana-Farber Cancer Institute, Harvard Medical School, Boston, MA, USA; ^4^ Mount Auburn Hospital, Harvard Medical School, Cambridge, MA, USA; ^*^ These authors contributed equally to this work

**Keywords:** multiple myeloma, inflammation mark

## Abstract

Elevated inflammatory markers are associated with poor outcomes in various types of cancers; however, their clinical significance in multiple myeloma (MM) have seldom been explored. This study investigated the prognostic relevance of neutrophil-to-lymphocyte ratio (NLR), platelet-to-lymphocyte ratio (PLR), and monocyte-to-lymphocyte ratio (MLR) in MM. Totally 559 MM patients were included in this study. NLR, PLR and MLR were calculated from whole blood counts prior to therapy. Kaplan-Meier curves and multivariate Cox proportional models were used for the evaluation of the survival. It has shown that newly diagnosed MM patients were characterized by high NLR and MLR. Elevated NLR and MLR and decreased PLR were associated with unfavorable clinicobiological features. Applying cut-offs of 4 (NLR), 100 (PLR) and 0.3 (MLR), elevated NLR, MLR and decreased PLR showed a negative impact on outcome. Importantly, elevated NLR and decreased PLR were independent prognostic factors for progression-free survival. Thus, elevated NLR and MLR, and decreased PLR predict poor clinical outcome in MM patients and may serve as the cost-effective and readily available prognostic biomarkers.

## INTRODUCTION

Multiple myeloma (MM) is a malignant tumor of plasma cells characterized by a strong dependence on bone marrow milieu [1]. The interaction between MM cells and bone marrow microenvironment plays a crucial role in MM pathogenesis. It is now becoming clear that MM microenvironment is largely orchestrated by inflammatory cells including macrophages, dendritic cells, mast cells and myeloid-derived suppressor cells [2–4]. These cells are the major sources of cytokines in MM-infiltrated bone marrow [5], and meanwhile they can mediate immune suppression in MM. Moreover, MM is characterized by high levels of CRP, IL-6, IGF-1, TGF-β and IL-17 as the markers of systemic inflammatory response [6]. These data suggest that a tight link exists between inflammation and pathogenesis in MM.

Inflammation is one of the hallmarks of cancer and tumor-associated inflammatory response has a critical role in enhancing tumorigenesis by inducing tumor cell growth, angiogenesis and genome instability [7]. The host response to malignant tumors consists of changes in the tumor microenvironment as well as systemic alterations. Systemic inflammation is associated with the alteration in peripheral blood leukocytes that can be captured by neutrophil-lymphocyte ratio (NLR). Recently, platelet-to-lymphocyte ratio (PLR) has been introduced as another measurable parameter to determine inflammation [8, 9]. Moreover, monocyte-to-lymphocyte ratio (MLR) has been proposed as an indicator of systemic inflammatory response [10, 11]. Importantly, these emerging biomarkers have revealed the impact on clinical outcome of several solid tumors, such as cervical carcinoma, kidney cancer, gastrointestinal cancer and lung cancer [8, 12–15]. However, the clinical significance of myeloma-associated inflammation is largely unknown.

It is widely recognized that the biological behavior of MM cells is determined by both their genetic background and their bone marrow microenvironment; however, the current risk stratification is mainly based on tumor biology, tumor burden, and patient characteristics. No factors related to microenvironment have been enrolled [16]. One of the reasons is the lack of easy-to-access prognostic biomarkers. Thus, the purpose of the present study was to evaluate the prognostic value of cellular components of the systemic inflammatory response, including of NLR, PLR and MLR on progression-free survival (PFS) and overall survival (OS) in MM patients.

## RESULTS

### Newly diagnosed MM patients were characterized by high NLR and MLR

**Figure 1:**
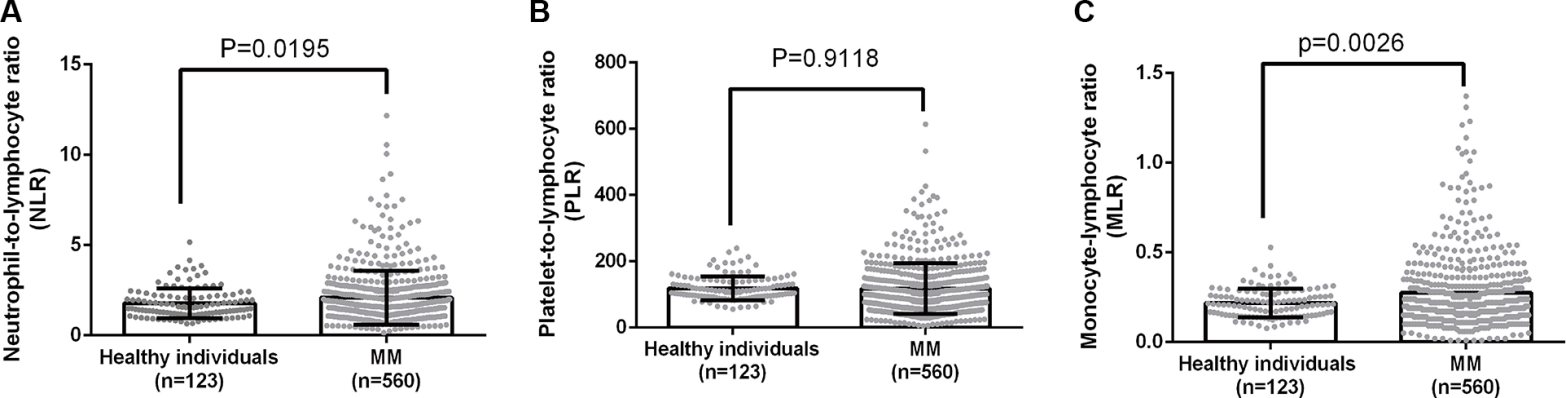
A comparison of NLR, PLR and MLR between healthy individuals and MM patients

We firstly analyzed NLR, MLR and PLR in 559 newly diagnosed MM patients. Totally 123 healthy individuals were included in the present study as the controls. The NLRs in healthy control and MM were 1.771 ± 0.07471 and 2.096 ± 0.06299 (*p* = 0.0195); the PLRs were 118.7 ± 3.263 and 117.9 ± 3.250 (*p* = 0.9118); and the MLRs were 0.2170 ± 0.007272 and 0.2739 ± 0.008684 (*p* = 0.0026) (Figure 1A–1C).

**Figure 2:**
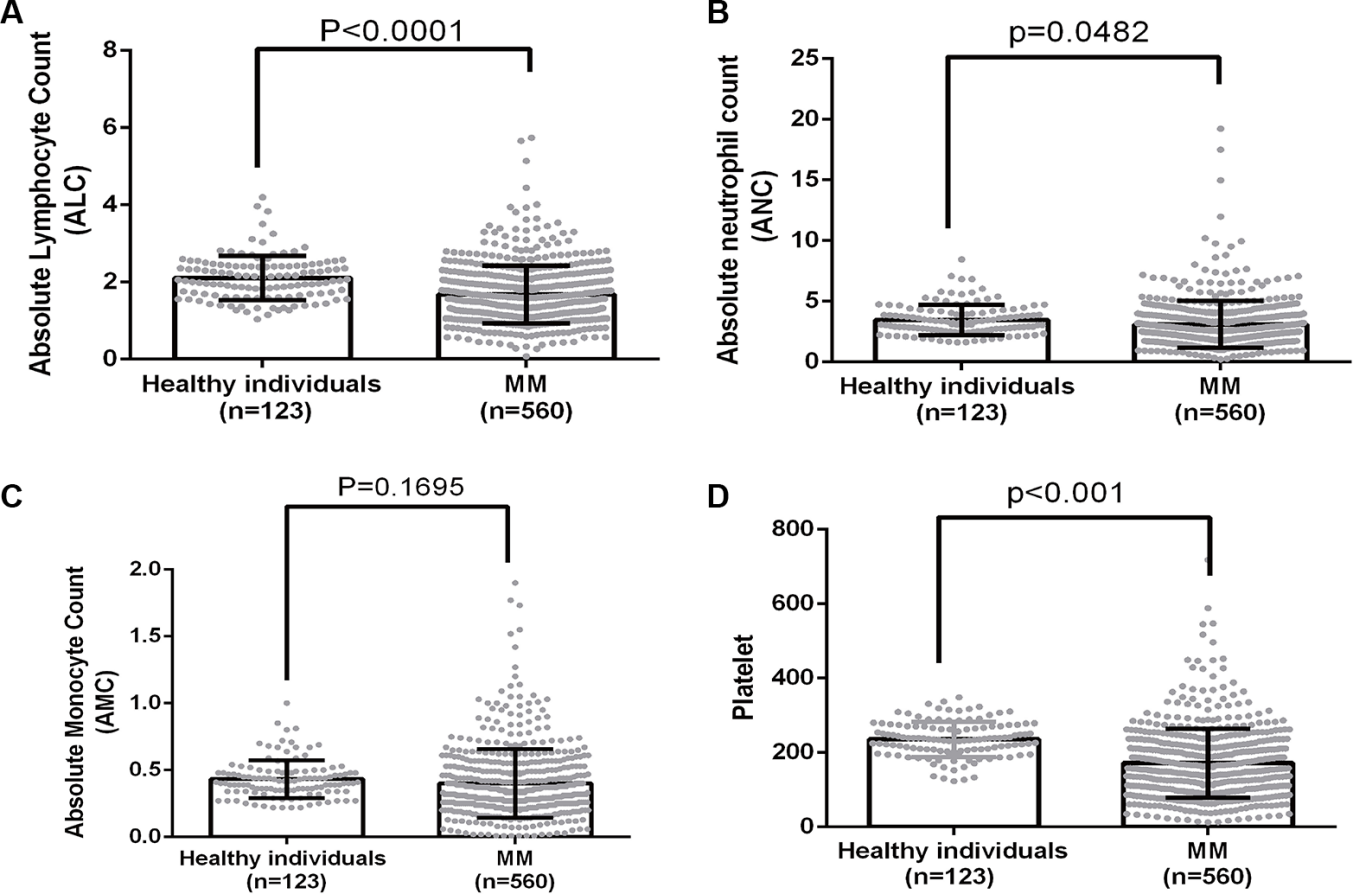
A comparison of ANC, ALC, AMC and platelet count between healthy individuals and MM patients

We further explored the causes of higher NLR and MLR in MM. As shown in Figure 2, ALC in healthy control and MM were 2.104 ± 0.05161 and 1.677 ± 0.03167 (*p <* 0.0001); ANC were 3.469 ± 0.1126 and 3.090 ± 0.08205 (*p* = 0.0482); AMC were 0.4321 ± 0.01267 and 0.3993 ± 0.01085 (*p* = 0.1695); platelet counts were 235.8 ± 4.268 and 171.4 ± 3.940 (*p <* 0.0001). These data suggested that MM selectively induced lymphopenia and thrombocytopenia, but myeloid cells, especially monocytes, behaved distinctly with a much less pronounced decline.

### Predictive effect of NLR, PLR and MLR

**Figure 3:**
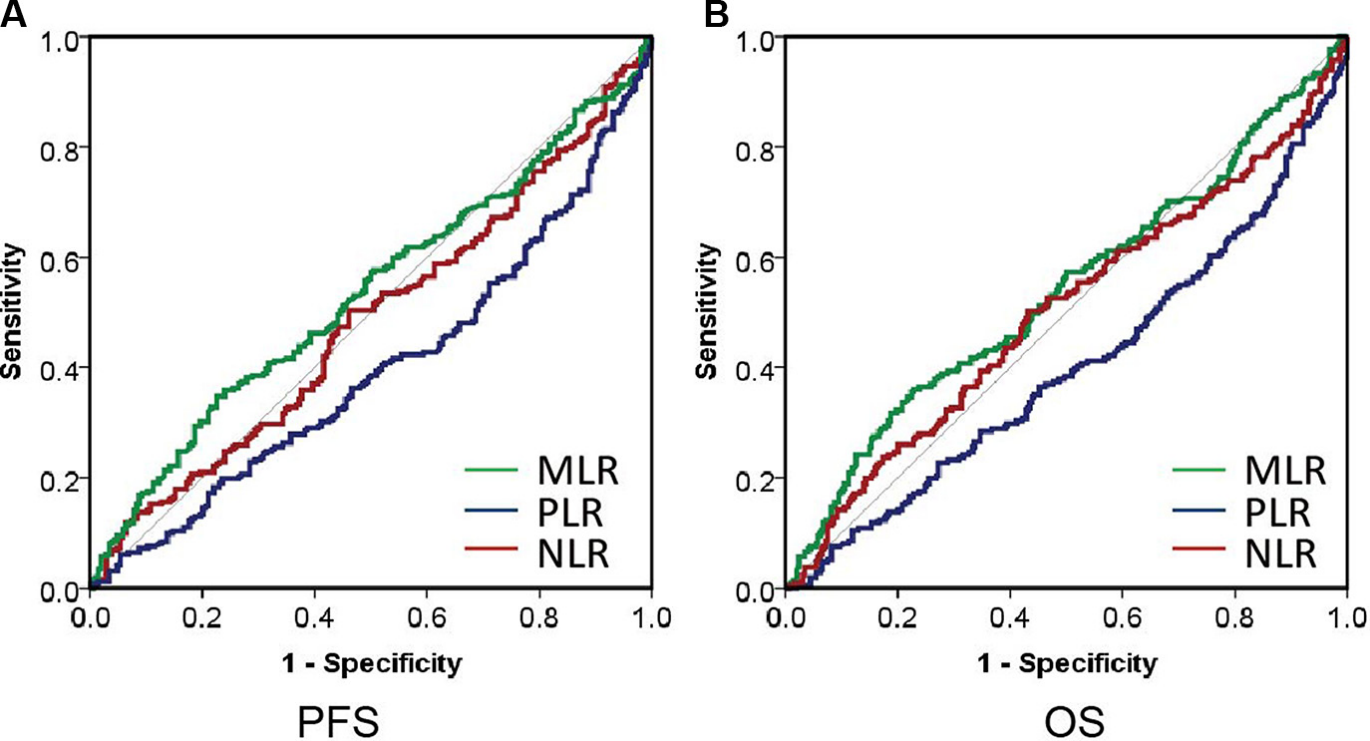
The ROC curve analysis for the optimal cutoff point of NLR, PLR and MLR

**Figure 4:**
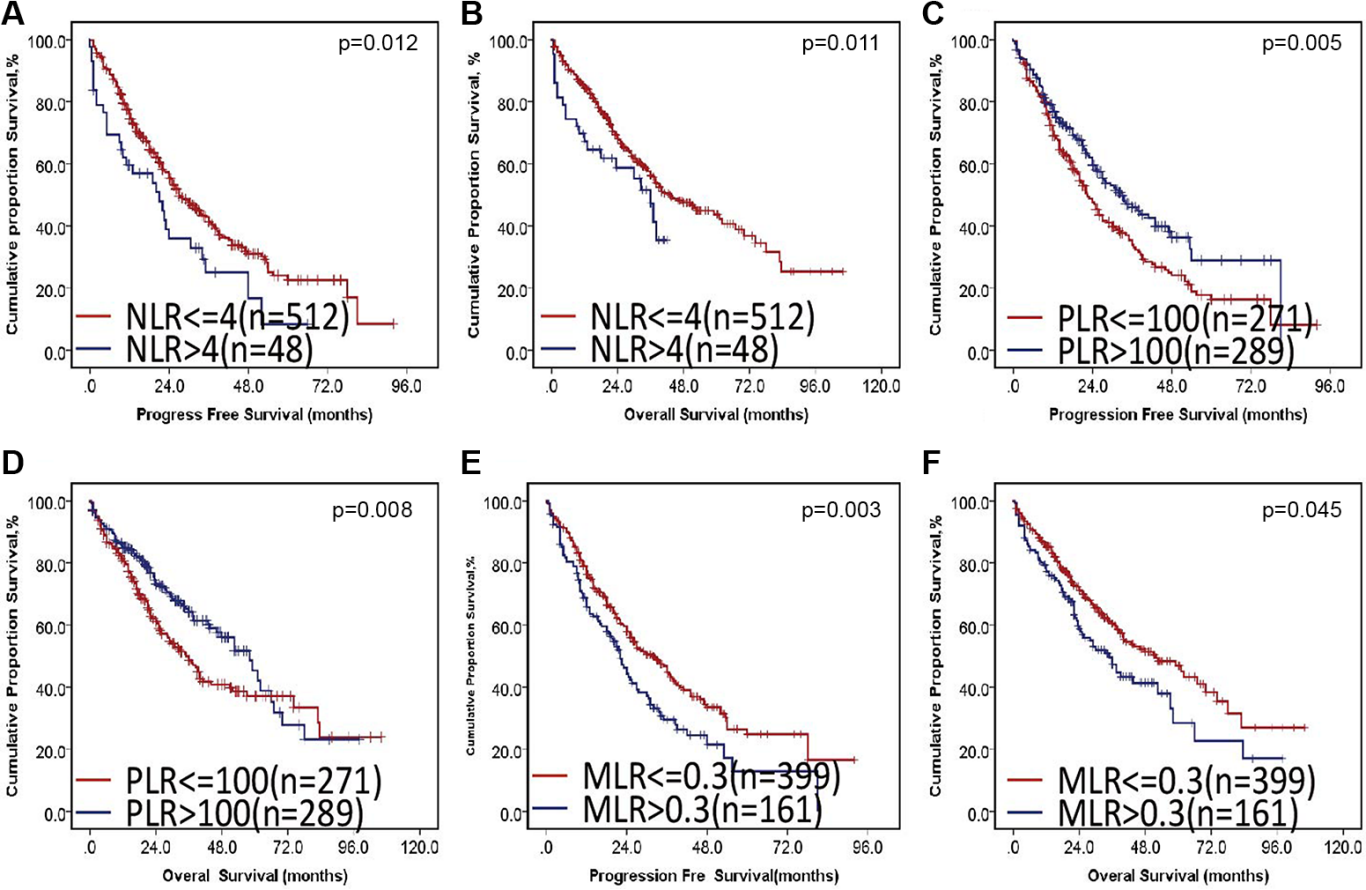
The impact of NLR, PLR and MLR on PFS and OS in MM

The cut-off values of NLR, PLR and MLR used for prognostication in cancers are not uniform in previous studies. We therefore performed cut-off optimization for NLR, PLR and MLR in our study cohort. We first analyzed the impact of NLR on the survival of MM. Cutoff points of 4.0 revealed the highest Youden value to progression and death in ROC curve (Figure 3A and 3B). We thus used 4.0 to group the patients into 2 categories. The median PFS was 24.03 months (95% CI: 17.1–31.0) in patients with high NLR and 37.46 months (95% CI: 33.6–41.3) in the rest of the cohort (*p* = 0.012, Figure 4A). These patients also had a significantly shortened OS, and the median OS was 43.2 (95% CI: 35.6–50.8) and 56.0 (95% CI: 49.9–62.1) months, respectively (*p* = 0.011, Figure 4B).

Next, we checked the impact of PLR on survival. Although PLR in healthy individual and MM was similar; however, PLR was tightly associated with progression and death, as shown in Figure 3. The median PLR in healthy control and MM was 110 and 102, respectively. We used 100 to group the patients into 2 categories. The median PFS was 32.3 months (95% CI: 27.9–36.8) in patients with decreased PLR and 40.4 months (95% CI: 35.3–45.5) in the rest of the cohort (*p* = 0.005, Figure 4C). These patients also had a significantly shortened OS, and the median OS was 49.4 (95% CI: 42.9–55.8) and 53.2 (95% CI: 46.8–59.7) months, respectively (*p* = 0.008, Figure 4D).

Finally, we studied the prognostic value of MLR. The cutoff point of 0.3 exhibited the highest Youden value to progression and death using ROC curve (Figure 3A and 3B). We used 0.3 to group the patients into 2 categories. The median PFS was 29.2 months (95% CI: 24.0–34.5) in patients with high NLR and 40.0 months (95% CI: 35.4–44.7) in the rest of the cohort (*p* = 0.003, Figure 4E). These patients also had a significantly shortened OS, and the median OS was 25.9 (95% CI: 25.9–141.0) and 53.7 (95% CI: 21.9–38.0) months, respectively (*p* = 0.045, Figure 4F).

### Multivariate analysis

A multivariate analysis including all parameters associated with PFS in the univariate analysis was performed. Chromosomal aberration with 17p deletion (HR 3.361 [95% CI: 2.057–5.492], *p <* 0.001), 1q21 gains (HR 2.260 [95% CI: 1.624–3.144], *p <* 0.001), t(4;14)(IgH/FGFR3) (HR 1.717[95% CI: 1.099–2.683], *p* = 0.018), elevated NLR (HR 2.468 [95% CI: 1.438–4.239], *p* = 0.001) and decreased PLR (HR 0.727 [95% CI: 0.524–1.108], *p* = 0.046) were statistically independent predictors of PFS.

A similar analysis for the prediction of OS identified the relevant parameters as follows: del(17p) (HR 2.865 [95% CI: 1.713–4.793], *p <* 0.001), 1q21 gains (HR 1.902 [95% CI: 1.310–2.763], *p* = 0.001) and ISS stage III (HR 1.766 [95% CI: 1.292–2.415], *p <* 0.001) (Supplementary Table S1).

### Elevated NLR, MLR and decreased PLR were associated with unfavorable clinico-pathological features

Next, we determined the associations of NLR, PLR and MLR with other clinical parameters. As shown in Table 1, patients with elevated NLR were characterized by high beta-2 microglobulin (median, 7.68 *vs*. 4.96 mg/L; *p* = 0.001) and ISS stage III (75.0% *vs*. 44.7%; *p <* 0.001). On the frequency of the type of M protein, IgD and free light chain MM were more common. The frequency of chromosome 13q deletion (29.7% *vs*. 47.3%, *p* = 0.0406) and t(4;14) (6.1% *vs*. 21.6%, *p* = 0.0339) was lower in patients with high NLR. Fewer patients with high NLR were identified as belonging to the high-risk cytogenetic group, but statistically significant difference was not reached (17.6% *vs*. 32.0%; *p* = 0.083).

**Table 1:** Demographics and baseline clinical characteristics evaluated by NLR

	NLR > 4 (*n* = 48 )	NLR < = 4 (*n* = 512)	*p*-value
**Characteristics**			
Male (%)	34 (70.8%)	310 (60.7%)	0.1710
Age (median, y)	62	58	0.4315
**Subtype of MM (%)**		0.0001	
IgG	17 (35.4%)	264 (51.6%)	
IgA	6 (12.5%)	127 (24.8%)	
IgD	5 (10.4%)	13 (2.5%)	
Light chain	18 (37.5%)	89 (17.4%)	
Non-secretory	2 (4.2%)	19 (3.7%)	
**ISS stage (%)**		< 0.001	
I	4 (8.3%)	96 (18.8%)	
II	8 (16.7%)	187 (36.5%)	
III	36 (75%)	229 (44.7%)	
**Durie-Salmon stage (%)**		0.1497	
I–II	4 (8.3%)	83 (16.2%)	
III	44 (91.6%)	429 (83.8%)	
**Other Parameters**			
Beta 2-microglobulin (median, mg/L)	7.68	4.96	0.001
Beta 2-microglobulin < 3.5 mg/L (%)	5 (10.4%)	64 (12.5%)	< 0.0001
Beta 2-microglobulin ≥ 5.5 mg/L (%)	36 (75%)	195 (38.1%)	< 0.0001
LDH (median, U/L)	185	150	0.0229
Hemoglobin (median, g/L)	90.0	85.0	0.7505
Albumin (median, g/L)	33.8	34.8	0.4419
Platelet count (median, × 10^9^/L)	176	157	0.0066
PCs by morphology (median, %)	19.25	29.0	0.1129
PCs by MFC (median, %)	5.73	7.79	0.9465
**Cytogenetic abnormalities (%)**			
del(13q)	11/37 (29.7%)	183/387 (47.3)	0.0406
del(17p)	3/37 (8.1%)	36/386 (9.3%)	0.8067
1q21 gains	12/34 (35.3%)	160/355 (45.1%)	0.2729
IGH translocations	16/36 (44.4%)	222/388 (57.2%)	0.1396
t(11;14)	5/35 (14.3%)	71/365 (19.5%)	0.4567
t(4;14)	2/33 (6.1%)	77/357 (21.6%)	0.0339
t(14;16)	2/33 (4.2%)	14/353 (4.0%)	0.5638
High-risk cytogenetics	6/34 (17.6%)	113/353 (32.0%)	0.0830

Note: High-risk cytogenetics were defined by the presence of t(4;14), t(14;16), and/or del(17p).

Table 2 contains data comparing clinico-pathological factors within the high and low PLR groups. Patients with decreased PLR were characterized with high tumor burden: higher frequency of elevated beta-2 microglobulin (median, 5.79 *vs*. 4.74 mg/L; *p <* 0.012), ISS stage III (54.2% *vs*. 40.8%; *p* = 0.0064), low hemoglobin (79.0 *vs*. 93.0 g/L; *p <* 0.0001), higher percentage of plasma cells as assessed by conventional morphology (31.5% *vs*. 25.5% PCs; *p* = 0.0003), or by immunophenotyping (9.38% *vs*. 5.75% PCs; *p* = 0.0197). The frequency of patients harboring 17p deletion detected by FISH was significantly higher in patients with decreased PLR (14.0% *vs*. 4.9%; *p* = 0.0013). There was a trend for higher incidence of 13q deletion and 1q gains, but statistically significant difference was not reached (50.5% *vs*. 41.5%, *p* = 0.0638; 49.2% *vs*. 39.8%, *p* = 0.0631). More patients with decreased PLR were identified as belonging to the high-risk cytogenetic group (37.8% *vs*. 24.3%; *p* = 0.0038). It was clear that decreased PLR was associated with a more advanced and aggressive tumor type.

**Table 2:** Demographics and baseline clinical characteristics evaluated by PLR

	PLR >100 (*n* = 289 )	PLR< = 100 (*n* = 271)	*p*-value
**Characteristics**			
Male (%)	174 (60.2%)	172 (63.5%)	0.4274
Age (median, y)	58	59	0.2566
**Subtype of MM (%)**			
IgG	147	129	0.1273
IgA	64	66	
IgD	8	10	
Light chain	44	63	
Non-secretory	8	16	
**ISS stage (%)**			0.0064
I	58 (20.1%)	42 (15.5%)	
II	113 (41.1%)	82 (30.3%)	
III	118 (40.8%)	147 (54.2%)	
**Durie-Salmon stage (%)**			0.0974
I–II	52 (18.0%)	35 (12.9%)	
III	237 (82.0%)	236 (87.1%)	
**Other Parameters**			
Beta 2-microglobulin (median, mg/L)	4.74	5.79	0.0012
Beta 2-microglobulin < 3.5 mg/L (%)	38 (13.1%)	31 (11.4%)	0.6852
Beta 2-microglobulin ≥ 5.5 mg/L (%)	99 (37.4%)	128 (51.6%)	0.0018
LDH (median, U/L)	152.0	151.0	0.1684
Hemoglobin (median, g/L)	93.0	79.0	< 0.0001
Albumin (median, g/L)	36.1	33.5	0.8060
Lymphocyte count (median, ×10^9^/L)	1.355	1.850	< 0.0001
PCs by morphology (median, %)	25.5	31.5	0.0003
PCs by MFC (median, %)	5.75	9.375	0.0197
**Cytogenetic abnormalities (%)**			
del(13q)	93/224 (41.5%)	101/200 (50.5%)	0.0638
del(17p)	11/223 (4.9%)	28/200 (14.0%)	0.0013
1q21 gains	82/206 (39.8%)	90/183 (49.2%)	0.0631
IGH translocations	112/219 (51.1%)	126/205 (46.5%)	0.0323
t(11;14)	36/207 (17.4%)	40/193 (14.8%)	0.3957
t(4;14)	35/206 (17.0%)	44/184 (23.9%)	0.0895
t(14;16)	7/204 (3.4%)	9/182 (4.9%)	0.4564
High-risk cytogenetics	49/202 (24.3%)	70/185 (37.8%)	0.0038

Note: High-risk cytogenetics were defined by the presence of t(4;14), t(14;16), and/or del(17p).

Patients with elevated MLR were characterized by high beta-2 microglobulin (median, 7.04 vs. 4.84 mg/L; *p <* 0.001) and ISS stage III (61.5% vs. 41.6%; *p <* 0.001). No association between MLR level and cytogenetic abnormality detected by FISH was observed (Supplementary Table S2).

## DISCUSSION

Although it is recognized that cancer formation has a genetic basis, there is increasing evidence that the host inflammatory response plays an important role in the development and progression of cancer. Chronic inflammation is one of the hallmarks of cancer and contributes to cancer development [17]. NLR, PLR and MLR are simple indicators of systemic inflammation, which can provide prognostic information in a wide range of cancer types. Herein, we find MM tumor cells selectively induced lymphopenia and thrombocytopenia, but have less impact on myeloid cells, especially monocytes. Newly diagnosed MM patients are characterized by high NLR and MLR. Elevated NLR and MLR and decreased PLR are associated with unfavorable clinico-pathological features and have a negative impact on survival of MM. Importantly, elevated NLR and decreased PLR have independent prognostic value on PFS. Our results have demonstrated that systemic inflammation markers can be valuable biomarkers for the prognosis and risk stratification of MM.

Anemia is a common clinical feature of MM. Thrombocytopenia and lymphopenia are also common when the tumor load is high. However, myeloid cells are usually normal in whole blood cell count. Indeed, our results have first shown a significantly lower ALC, ANC and platelet count when compared with healthy individuals, whereas AMC is similar between MM and healthy control. Our data show that MM cells cannot simply displace hematopoietic cells upon BM infiltration, but rather selectively modulate the BM microenvironment.

Chronic inflammation contributes to cancer development via multiple mechanisms. One potential mechanism is that chronic inflammation can generate an immunosuppressive microenvironment for tumor formation and progression [18]. Monocyte-derived cells are very important for both MM survival and immune escape. Especially, various monocyte-derived cells, including macrophages, myeloid-derived suppressor cells (MDSCs) and dendritic cells (DCs), play an important role in mediating immune suppression in MM [3, 4, 19]. These cells are recruited by MM cells to create a localized immunosuppressive microenvironment for MM survival, which could be that reasons that MM patients have a normal monocyte level regardless of BM infiltration. Monocytes may represent a novel mechanism by which the pro-inflammatory response is linked to immune tolerance in the tumor milieu. Indeed, activated monocytes in hepatocellular carcinoma microenvironment foster immune privilege and disease progression through PD-L1 [20], which may be the reason that MM cells selectively preserve a pool of myeloid precursor cells.

We have further confirmed that MM patients have significantly elevated NLR and MLR, whereas, PLR is similar between healthy individuals and MM patients. Lymphocytes have an important role in immune surveillance. ALC has been identified as a surrogate marker of the host anti-tumor immune status, and has been demonstrated as an independent prognostic parameter for predicting survival and clinical outcome in a number of hematological malignancies. Indeed, our study has demonstrated a significantly decreased ALC in MM and the decline of ALC leads to the elevated NLR and MLR.

The elevated NLR has been reported to be a poor prognostic indicator in several malignancies [21], but only few reports have examined its impact on MM [22, 23]. Consistent with precious study, our results have demonstrated that elevated NLR was associated with unfavorable clinico- pathological features. Especially, elevated NLR was associated with high beta-2 microglobulin, but low frequency of high-risk cytogenetic abnormalities. These data suggested that NLR was an inflammatory marker and its impact on survival was independent on cytogenetic abnormalities. However, different cut-off values were used due to different patients and treatments in several series recently published. Moreover, the prognostic value of NLR may be more important in patients receiving immunomodulatory drugs (IMiDs) or immunotherapy. Larger cohorts and prospective studies are needed to establish the cut-off and prognostic value of NLR.

Emerging evidence strongly supports that platelets are also part of the inflammatory response and thrombocytosis is common in patients with solid tumors [24]. In addition, PLR is introduced as a potential marker to determine inflammation, and a higher platelet to lymphocyte ratio is associated with poor prognosis in a few types of cancer [25]. MM patients show evidence of platelet activation as measured by elevated plasma soluble P-selectin [26] and are highly susceptible to therapy-induced thrombosis [27]. Importantly, activated platelets secrete many cytokines that are required for growth of myeloma cells including IL-6, VEGF, SDF-1α, and IGF-1, suggesting that platelets may affect the microenvironments of MM. However, with the expansion of malignant cells in bone marrow, thrombocytopenia is frequently observed in MM. Thus, PLR is not only an inflammatory marker, but also a marker of tumor burden and aggressiveness in MM. Indeed, we have found that PLR is associated with high tumor burden parameters in this study, and importantly, more patients belong to the high-risk cytogenetic group.

MM is a plasma cell malignancy critically dependent on signals generating from the inflammatory microenvironment for survival and proliferation. Inflammatory factors may play a critical role in risk stratification of MM. Our study indicated that elevated NLR, MLR and decreased PLR were associated with poor prognosis in MM. Importantly, elevated NLR and decreased PLR were independent prognostic factors. However, multivariate analysis showed these inflammation-related factors were only loosely and independently prognostic for OS. These data suggested that NLR and PLR were low-cost and easy-to-access tests that predicted PFS in MM. But cytogenetic aberration and ISS stages are still the most important prognostic factors of OS in MM.

The strengths of the current study include its large patient cohort and comparatively long follow-up period. There are also limitations in this study. NLR, MLR and PLR have been widely used to evaluate the inflammation of solid tumor; however, MM is a tumor in bone marrow and bone marrow infiltration should been taken into account. Unlike diffused bone infiltration in leukemia, MM bone marrow infiltration is patchy; however, it obviously impairs hematopoiesis at least when the tumor load is high enough. Thus, NLR, MLR and PLR reflect not only inflammation, but also bone marrow infiltration. Indeed, we find high population of plasma cells in bone marrow with decreased PLR.

In summary, the results of the present study showed that NLR, PLR and MLR were prognostic in patients with MM. They could be used as the easily accessible and reliable markers to predict MM prognosis.

## MATERIALS AND METHODS

### Study population

The myeloma patients were enrolled from a prospective, nonrandomized clinical trial (BDH 2008/02) as previously described [28]. The trial was conducted in accordance with the Declaration of Helsinki (Version 1996) and approved by the local ethics committees of institutions. The diagnostic criteria were consistent with the criteria for symptomatic MM as defined and published by the International Myeloma Working Group [29]. The responses were evaluated using the International Myeloma Working Group criteria [30]. A cohort of 560 consecutive patients was enrolled between January 2008 and October 2013, with a median follow-up time of 64 months from diagnosis.

### Calculation of NLR, PLR and MLR

White blood cell (WBC), absolute neutrophil count (ANC), absolute lymphocyte count (ALC), absolute monocyte count (AMC) and platelet count were obtained from a standard CBC obtained at the time of diagnosis by Sysmex XN-9000/5000. The NLR was calculated as the ratio between absolute neutrophil count and absolute lymphocyte count. The PLR was calculated as the ratio between absolute platelet count and absolute lymphocyte count. MLR was calculated as the ratio between absolute monocyte count and absolute lymphocyte count. Receiver-operating characteristic curve (ROC) analysis was used to identify the optimal value of NLR, PLR and MLR in relation to progression-free survival (PFS) and overall survival (OS).

### FISH study

All MM cell samples were purified using Miltenyi technology (anti-CD138-coated magnetic beads; Paris, France) before FISH as reported previously [31]. Plasma cells were then analyzed using DNA probes specific for the following chromosomal aberrations: 13q14 deletion, 17p13 deletion, t(11;14), t(4;14), and t(14;16). Gains of 1q21 were assessed using a bacterial artificial chromosome probe at 1q21 (RP11-307C12) [32]. A total of 200 interphase nuclei were analyzed. According to Intergroupe Francophone du Myélome (IFM), high-risk MM was defined as the presence of any one or more of the following genetic abnormalities: deletion of 17p13, t(4;14), and t(14;16) [31].

### Statistical analysis

The primary end point was correlated with the survival from the time of diagnosis. PFS was calculated from the start point of the treatment to disease progression or the date of death (regardless of cause of death), whichever came first. OS was measured from the initiation of treatment to the date of death or last follow-up, according to the international uniform response criteria [33]. Unpaired *T* tests were used to assess the associations between categorical variables, with a confidence coefficient of 95%. The survival curves were plotted using the Kaplan-Meier method, with difference assessed by the log-rank test. The *P-value* of less than or equal to 0.05 was considered statistically significant.

ROC analysis was applied to determine the optimal cutoff value yielded the greatest differential in survival. The Youden's index was used to assess the optimum cutoff point for NLR, PLR and MLR [34]. The cutoff value with best discrimination (in mean of sensitivity and specificity) between survival and death was used for OS, and the cutoff value with best discrimination between progression and no progression was used for PFS. The Graphpad prism was used to conduct unpaired *T* test and SPSS 21.0 was used to perform survival analysis.

## SUPPLEMENTARY MATERIALS TABLES



## References

[1] Palumbo A, Anderson K (2011). Multiple myeloma. N Engl J Med.

[2] Chauhan D, Singh AV, Brahmandam M, Carrasco R, Bandi M, Hideshima T, Bianchi G, Podar K, Tai YT, Mitsiades C, Raje N, Jaye DL, Kumar SK (2009). Functional interaction of plasmacytoid dendritic cells with multiple myeloma cells: a therapeutic target. Cancer Cell.

[3] Gorgun GT, Whitehill G, Anderson JL, Hideshima T, Maguire C, Laubach J, Raje N, Munshi NC, Richardson PG, Anderson KC (2013). Tumor-promoting immune-suppressive myeloid-derived suppressor cells in the multiple myeloma microenvironment in humans. Blood.

[4] Zheng Y, Cai Z, Wang S, Zhang X, Qian J, Hong S, Li H, Wang M, Yang J, Yi Q (2009). Macrophages are an abundant component of myeloma microenvironment and protect myeloma cells from chemotherapy drug-induced apoptosis. Blood.

[5] Tai YT, Anderson KC (2015). Targeting B-cell maturation antigen in multiple myeloma. Immunotherapy.

[6] Prabhala RH, Pelluru D, Fulciniti M, Prabhala HK, Nanjappa P, Song W, Pai C, Amin S, Tai YT, Richardson PG, Ghobrial IM, Treon SP, Daley JF (2010). Elevated IL-17 produced by TH17 cells promotes myeloma cell growth and inhibits immune function in multiple myeloma. Blood.

[7] Hanahan D, Weinberg RA (2011). Hallmarks of cancer: the next generation. Cell.

[8] Krenn-Pilko S, Langsenlehner U, Thurner EM, Stojakovic T, Pichler M, Gerger A, Kapp KS, Langsenlehner T (2014). The elevated preoperative platelet-to-lymphocyte ratio predicts poor prognosis in breast cancer patients. British journal of cancer.

[9] Yan M, Jurasz P (2016). The role of platelets in the tumor microenvironment: From solid tumors to leukemia. Biochimica et biophysica acta.

[10] Cummings M, Merone L, Keeble C, Burland L, Grzelinski M, Sutton K, Begum N, Thacoor A, Green B, Sarveswaran J, Hutson R, Orsi NM (2015). Preoperative neutrophil: lymphocyte and platelet: lymphocyte ratios predict endometrial cancer survival. British journal of cancer.

[11] Li J, Jiang R, Liu WS, Liu Q, Xu M, Feng QS, Chen LZ, Bei JX, Chen MY, Zeng YX (2013). A large cohort study reveals the association of elevated peripheral blood lymphocyte-to-monocyte ratio with favorable prognosis in nasopharyngeal carcinoma. PLoS One.

[12] Absenger G, Szkandera J, Pichler M, Stotz M, Arminger F, Weissmueller M, Schaberl-Moser R, Samonigg H, Stojakovic T, Gerger A (2013). A derived neutrophil to lymphocyte ratio predicts clinical outcome in stage II and III colon cancer patients. British journal of cancer.

[13] Ishizuka M, Nagata H, Takagi K, Iwasaki Y, Kubota K (2013). Combination of platelet count and neutrophil to lymphocyte ratio is a useful predictor of postoperative survival in patients with colorectal cancer. British journal of cancer.

[14] Kang MH, Go SI, Song HN, Lee A, Kim SH, Kang JH, Jeong BK, Kang KM, Ling H, Lee GW (2014). The prognostic impact of the neutrophil-to-lymphocyte ratio in patients with small-cell lung cancer. British journal of cancer.

[15] Kinoshita A, Onoda H, Imai N, Iwaku A, Oishi M, Fushiya N, Koike K, Nishino H, Tajiri H (2012). Comparison of the prognostic value of inflammation-based prognostic scores in patients with hepatocellular carcinoma. British journal of cancer.

[16] Mikhael JR, Dingli D, Roy V, Reeder CB, Buadi FK, Hayman SR, Dispenzieri A, Fonseca R, Sher T, Kyle RA, Lin Y, Russell SJ, Kumar S (2013). Management of Newly Diagnosed Symptomatic Multiple Myeloma: Updated Mayo Stratification of Myeloma and Risk-Adapted Therapy (mSMART) Consensus Guidelines 2013. Mayo Clin Proc.

[17] Crusz SM, Balkwill FR (2015). Inflammation and cancer: advances and new agents. Nat Rev Clin Oncol.

[18] Wang D, DuBois RN (2015). Immunosuppression associated with chronic inflammation in the tumor microenvironment. Carcinogenesis.

[19] Leone P, Berardi S, Frassanito MA, Ria R, De Re V, Cicco S, Battaglia S, Ditonno P, Dammacco F, Vacca A, Racanelli V (2015). Dendritic cells accumulate in the bone marrow of myeloma patients where they protect tumor plasma cells from CD8+ T-cell killing. Blood.

[20] Kuang DM, Zhao Q, Peng C, Xu J, Zhang JP, Wu C, Zheng L (2009). Activated monocytes in peritumoral stroma of hepatocellular carcinoma foster immune privilege and disease progression through PD-L1. The Journal of experimental medicine.

[21] Templeton AJ, McNamara MG, Seruga B, Vera-Badillo FE, Aneja P, Ocana A, Leibowitz-Amit R, Sonpavde G, Knox JJ, Tran B, Tannock IF, Amir E (2014). Prognostic role of neutrophil-to-lymphocyte ratio in solid tumors: a systematic review and meta-analysis. Journal of the National Cancer Institute.

[22] Kelkitli E, Atay H, Cilingir F, Guler N, Terzi Y, Ozatli D, Turgut M (2014). Predicting survival for multiple myeloma patients using baseline neutrophil/lymphocyte ratio. Ann Hematol.

[23] Romano A, Parrinello NL, Consoli ML, Marchionni L, Forte S, Conticello C, Pompa A, Corso A, Milone G, Di Raimondo F, Borrello I (2015). Neutrophil to lymphocyte ratio (NLR) improves the risk assessment of ISS staging in newly diagnosed MM patients treated upfront with novel agents. Ann Hematol.

[24] Franco AT, Corken A, Ware J (2015). Platelets at the interface of thrombosis, inflammation, and cancer. Blood.

[25] Baranyai Z, Krzystanek M, Josa V, Dede K, Agoston E, Szasz AM, Sinko D, Szarvas V, Salamon F, Eklund AC, Szallasi Z, Jakab F (2014). The comparison of thrombocytosis and platelet-lymphocyte ratio as potential prognostic markers in colorectal cancer. Thrombosis and haemostasis.

[26] Lemancewicz D, Bolkun L, Mantur M, Semeniuk J, Kloczko J, Dzieciol J (2014). Bone marrow megakaryocytes, soluble P-selectin and thrombopoietic cytokines in multiple myeloma patients. Platelets.

[27] Falanga A, Marchetti M (2009). Venous thromboembolism in the hematologic malignancies. J Clin Oncol.

[28] An G, Li Z, Tai YT, Acharya C, Li Q, Qin X, Yi S, Xu Y, Feng X, Li C, Zhao J, Shi L, Zang M (2015). The impact of clone size on the prognostic value of chromosome aberrations by fluorescence in situ hybridization in multiple myeloma. Clin Cancer Res.

[29] IMWG (2003). Criteria for the classification of monoclonal gammopathies, multiple myeloma and related disorders: a report of the International Myeloma Working Group. Br J Haematol.

[30] Rajkumar SV, Harousseau JL, Durie B, Anderson KC, Dimopoulos M, Kyle R, Blade J, Richardson P, Orlowski R, Siegel D, Jagannath S, Facon T, Avet-Loiseau H (2011). Consensus recommendations for the uniform reporting of clinical trials: report of the International Myeloma Workshop Consensus Panel 1. Blood.

[31] Avet-Loiseau H, Attal M, Moreau P, Charbonnel C, Garban F, Hulin C, Leyvraz S, Michallet M, Yakoub-Agha I, Garderet L, Marit G, Michaux L, Voillat L (2007). Genetic abnormalities and survival in multiple myeloma: the experience of the Intergroupe Francophone du Myelome. Blood.

[32] Hanamura I, Stewart JP, Huang Y, Zhan F, Santra M, Sawyer JR, Hollmig K, Zangarri M, Pineda-Roman M, van Rhee F, Cavallo F, Burington B, Crowley J (2006). Frequent gain of chromosome band 1q21 in plasma-cell dyscrasias detected by fluorescence in situ hybridization: incidence increases from MGUS to relapsed myeloma and is related to prognosis and disease progression following tandem stem-cell transplantation. Blood.

[33] Anderson KC, Kyle RA, Rajkumar SV, Stewart AK, Weber D, Richardson P (2008). Clinically relevant end points and new drug approvals for myeloma. Leukemia.

[34] Fluss R, Faraggi D, Reiser B (2005). Estimation of the Youden Index and its associated cutoff point. Biometrical journal Biometrische Zeitschrift.

