# Supplementary material for: Elevated neutrophil-to-lymphocyte ratio and monocyte-to-lymphocyte ratio and decreased platelet-to-lymphocyte ratio are associated with poor prognosis in multiple myeloma
Source: Oncotarget. 2016 Nov 12;8(12):18792–801. doi: 10.18632/oncotarget.13320 (PMC5386647; doi:10.18632/oncotarget.13320)
Supplement: Supplementary file 1 [file oncotarget-08-18792-s001.pdf]

Elevated neutrophil-to-lymphocyte ratio and monocyte-to-lymphocyte ratio and decreased platelet-to-lymphocyte ratio are associated with poor prognosis in multiple myeloma

Supplementary Materials

Supplementary Table S1: Results of Cox regression analysis of PFS and OS time from diagnosis

Prognostic parameter	Hazard ratio for PFS (95% CI)	*P*	Hazard ratio for OS (95% CI)	*P*
elevated NLR(NLR > 4)	2.468 (1.438–4.239)	0.001	0.313 (0.728–2.697)	0.313
decreased PLR(PLR < 100)	0.727 (0.524–1.108)	0.046	0.718 (0.493–1.047)	0.085
17p deletion	3.361 (2.057–5.492)	< 0.001	2.865 (1.713–4.793)	< 0.001
1q21 gains	2.260 (1.624–3.144)	< 0.001	1.902 (1.310–2.763)	0.001
13q deletion	1.112 (0.758–1.632)	0.586	1.128 (0.738–1.725)	0.576
t(11;14)(IgH/CCND1)	1.426 (0.939–2.167)	0.096	1.496 (0.940–2.381)	0.089
t(4;14)(IgH/FGFR3)	1.717 (1.099–2.683)	0.018	1.377 (0.837–2.268)	0.208
t(14;16)(IgH/MAF)	1.610 (0.792–3.273)	0.188	0.938 (0.397–2.215)	0.884
ISS stage III	1.218 (0.947–1.565)	1.218	1.766 (1.292–2.415)	< 0.001
DS stage III	1.218 (0.817–1.816)	0.333	1.203 (0.716–2.023)	0.485

Supplementary Table S2: Demographics and baseline clinical characteristics evaluated by MLR

	MLR > 0.3 (*n* = 161)	MLR < = 0.3 (*n* = 399)	*p*-value
**Characteristic**			
Male (%)	161 (72.0%)	230 (57.5%)	< 0.001
Age (median, y)	61.0	58.0	0.0624
**Subtype of MM (%)**	0.0016		
IgG	67 (41.6%)	217 (54.4%)	
IgA	34 (21.1%)	96 (24.1%)	
IgD	10 (6.2%)	8 (2%)	
Light chain	43 (26.7%)	64 (16.0%)	
Nonsecretory	7 (4.3%)	14 (3.5%)	
**ISS stage (%)**	< 0.001		
Ⅰ	22 (13.7%)	78 (19.5%)	
Ⅱ	40 (24.8%)	155 (38.8%)	
Ⅲ	99 (61.5%)	166 (41.6%)	
**Durie-Salmon stage (%)**	0.0389		
Ⅰ - Ⅱ	17 (10.5%)	70 (17.5%)	
Ⅲ	144 (89.5%)	329 (82.5%)	
**Other Parameters**			
Beta 2-microglobulin (median, mg/L)	7.04	4.84	< 0.0001
Beta 2-microglobulin < 3.5 mg/L (%)	2 (1.24%)	21 (5.3%)	0.03
Beta 2-microglobulin ≥ 5.5 mg/L (%)	102 (63.4%)	172 (43.1%)	< 0.001
LDH (median, U/L)	175.5	146.0	< 0.001
Hemoglobin (median, g/L)	84.0	86.0	0.1607
Albumin (median, g/L)	36.1	34.4	0.1345
Platelet count (median, ×109/L)	149	165	0.3355
PCs by morphology (median, %)	29.5	28.25	0.9439
PCs by MFC (median, %)	8.39	7.30	0.6774
**Cytogenetic abnormalities (%)**			
del(13q)	67/129 (51.9%)	127/295 (43.1%)	0.3395
del(17p)	16/127 (12.6%)	23/296 (7.8%)	0.1157
1q21 gains	60/117 (51.3%)	112/272 (41.2%)	0.0657
IGH translocations	74/130 (56.9%)	164/294 (55.8%)	0.8272
t(11;14)	23/122 (18.9%)	53/278 (19.1%)	0.9603
t(4;14)	17/116 (14.7%)	62/274 (22.6%)	0.0733
t(14;16)	8/115 (7%)	8/271 (3%)	0.0711
High-risk cytogenetics	36/116 (31.0%)	83/271 (30.6%)	0.9366

Note: High-risk cytogenetics were defined by the presence of t(4;14), t(14;16), and/or del(17p).
